# Surgery

**Published:** 1861-09

**Authors:** John H. Packard

**Affiliations:** Philadelphia


OR, BI-MONTHLY NOVELTIES IN MEDICAL, PATHOLOGICAL, SURGICAL, OBSTETRICAL PHYSIOLOGICAL, AND CHEMICAL SCIENCE.

### SURGERY.

BY JOHN H. PACKARD, M.D., OF PHILADELPHIA.

I.—*Statistics of Operations for Strangulated Hernia at St. George's Hospital during Seven Years.* By G. F. COOPER and T. HOLMES, formerly Surgical Registrars to the Hospital. (Medical Times and Gazette, June 15, 1861.)

THE cases operated on were 121 in number, 33 of the inguinal, 85 of the femoral, and 3 of the umbilical variety; the total number of deaths was 37, or 30·5 per cent.

INGUINAL.—Total, 33; common oblique, 19; congenital, 10; infantile, 1; direct, 3; of these, 22 were on the right, and 11 on the left side; 9 died, or 27·7 per cent.

Of the 19 cases of common oblique hernia, 2 were females and the rest males; 4 died, or 21·05 per cent., of which deaths 1 was the result of bronchitis; 14 were on the right side, and 5 on the left.

Of the 10 congenital cases, (all males,) 7 were on the right side and 3 on the left; 4 died, or 40 per cent., 1 probably as the direct result of injuries received during taxis;* 3 were infants, 3 months, 7 months, and 14 months old respectively; of these, two died. One aged 14, where the hernia had existed since birth, died. The following recovered: 1 aged 24, where the hernia had existed since the age of 9 years. One aged 33, where the hernia had existed as long as he could remember. One aged 33, where the hernia had existed since the age of 6 years. One aged 54; of this case there was no history. One aged 58, where the hernia had existed 4 years only. Finally, the oldest patient in this class died after operation; he was 60 years of age; the hernia seems to have existed congenitally, and had been operated on 15 years previously.

It thus appears that in most of these cases the hernia was either known to be congenital, or to have existed for so long a period that it might fairly be presumed to have been so.

Of the 3 cases of direct hernia, there were 2 males and 1 female; 2 were on the left side and 1 on the right; 1 died, or 33·3 per cent.

In the case of infantile hernia, the patient was 32 years of age; the hernia had been known to exist for 20 years; it was on the left side. The patient recovered.

*Age of Patients with Strangulated Inguinal Hernia.*—The age of the pa-

* No doubt the principal, if not the sole reason for the greater mortality in this class of cases, is the large number of infants contained in it.

tients with congenital hernia is stated above. Of the 23 instances of the other forms of inguinal hernia, one was an infant 11 weeks old, (died of bronchitis,) and another 4 years. The youngest age of the remaining 21 cases was 15 years and the oldest 70. The occurrence of strangulation at the middle period of life is shown by the following statement of the ages of these 21 patients:—

| | | |
|---|---|---|
| From childhood to 30 years (inclusive) | . . . . . . | 4 |
| From 30 to 40 years " | . . . . . . . | 5—1 died. |
| From 40 to 50 " " | . . . . . . . | 6 |
| From 50 to 60 " " | . . . . . . . . | 4—3 died. |
| From 60 to 70 " " | . . . . . . . | 2 |

As to the length of time that this form of hernia had existed before the occurrence of strangulation little need be said beyond this, that in 2 cases only was the hernia quite recent. It seems useless to tabulate the "average duration" of the hernia previous to strangulation, since this depends so much on accidental circumstances.

*Period of Strangulation.*—Under this head the list of inguinal hernia gives a very favorable return, since the great majority were operated on within 24 hours of strangulation.

| | | | |
|---|---|---|---|
| 1st day of strangulation, | 19 | . . . | 3 died—all of peritonitis. In 1 it had begun before the operation; 1 of the others was an infant. |
| 2d " " | 5 | . . . | 2 died—1 (an infant) of exhaustion already begun; the other from phagedæna around an artificial anus. |
| 3d " " | 5 | . . . | 3 died—in all, the gut was irretrievably damaged; in 1 case by ulceration, and in the other 2 by forcible taxis, applied before admission. |
| 4th and 5th " | 4 | . . . | 1 died—of bronchitis, (an infant.) |

These figures clearly show how successful the operation for inguinal hernia may be expected to be in the adult, if the case be operated on before the occurrence of peritonitis; since, excluding from the above list the infants, the fatal case in which peritonitis had set in before the operation, and the 3 cases in which injury had been inflicted on the gut by the negligence or violence of the medical attendant, we have 20 cases and only 2 deaths; and as one of these was a very unusual and exeeptional instance of sloughing around an artificial anus, it would perhaps be more correct to say that out of 25 ordinary cases, in which the operation was performed early, only 1 died, and in this case the stricture was unusually tight, and the operation difficult from some folding or invagination at the neck of the sac.

*Trusses worn or not.*—It has been already stated that it is very rare for inguinal hernia to become speedily strangulated. Hence it is probable that if a well-fitting truss were applied soon after the first appearance of the tumor, few cases would ever require operation. In the only 2 in which the hernia was recent, no truss had been worn. Of the whole 33 cases, in 12 no truss had been worn; 2 had originally used trusses, but the instruments had soon become broken and had not been replaced; 4 others had exposed themselves to still further risk by wearing badly-fitting trusses; and in the other 15 cases a truss had been worn, but in many it was known not to have been adopted till long after the first occurrence of the hernia, and this was the case, doubtless, in several others; in whom, in consequence of the lapse of time, the circumstance had escaped their recollection.

*Contents of the Sac and their condition.**—In one case the sac was not opened. The sac in almost all cases contained serous fluid, varying in quantity as well as in the proportion of blood mixed with it. The latter seemed due, sometimes at least, to violence in the employment of the taxis.

The visceral contents of the 32 herniæ may be thus classified:—

| | | |
|---|---|---|
| Healthy or only congested small intestine . . . | 14—2 | deaths. |
| Inflamed small intestine . . . . . . . . . | 6—3 | " |
| Healthy small intestine with omentum . . . . | 9—2 | " |
| Inflamed large intestine . . . . . . . . . | 1—1 | " |
| Both large and small intestine with omentum . . | 2—1 | " |
| | 32—9 | deaths, |
| | | or 28·1 per cent. |

In many of the cases of entero-epiplocele, the omentum was adherent, thickened, or showed some trace of morbid action; in most of these it was excised, but as this fact does not seem to have affected the progress of the case, it will not be worth while to dwell particularly on the state of the omentum. In none of the cases of entero-epiplocele was the gut visibly inflamed, nor was the mortality much greater than in simple enterocele, and in one of the fatal cases, excessive taxis had been employed. In no instance was the gut found ulcerated in the line of stricture.

The three cases in which the large intestine was contained in the sac were all on the right side; in one, the appendix cæci was so riddled by ulceration that it was thought right to tie and cut it off. The other fatal case was that alluded to in which phagedæna attacked the margins of an artificial anus.

*Causes of Death.*—Hence it appears that the causes of death were in most of the cases entirely independent of the operation. We may also conclude that inguinal hernia is little liable to strangulation,† especially if early treated by a well-fitting truss; and that when strangulated, if the taxis fails, an operation affords every prospect of success, if early performed on a healthy patient.

UMBILICAL.—All the patients were females, and past middle life. The sac in each contained a good deal of omentum, which in one instance formed the stricture. The fatal result of one case was due to previous peritonitis.

FEMORAL.—This form of hernia is much more frequently strangulated than

* The data in regard to the significance and quantity of the fluid generally found in the sac were not sufficiently accurate in this set of cases, as recorded, to justify any deductions.

† It must be remembered that inguinal hernia is more than seven times as common as femoral, while its strangulation is hardly more than one third as common. See a paper by Mr. Bryant in *Guy's Hospital Reports*, Third Series, vol. ii.

inguinal. In the present table there are 85 of the former to 33 of the latter. Of the 85, 14 were males and 71 females; 46 had the right side affected, 37 the left, while in 2 cases this point was not noted. The deaths were 27, or 31·76 per cent.

*Age.*—One case occurred in a girl aged eight; all the rest were over twenty, as follows :—

| | | |
|---|---|---|
| From 20 to 30, inclusive . . . . . . . . . | 4 cases— | 1 died. |
| " 30 " 40 " . . . . . . . . . | 20 " | 3 " |
| " 40 " 50 " . . . . . . . . . | 21 " | 4 " |
| " 50 " 60 " . . . . . . . . . | 20 " | 7 " |
| " 60 " 80 " . . . . . . . . . | 19 " | 12 " |

Hence strangulation seems to occur in about the same number of cases in each decade after the age of 20, but to be more dangerous as life advances.

*Duration.*—In 20 cases the hernia was quite recent; so that in almost a quarter of the whole number strangulation commenced very soon, often, the patients thought, directly after the tumor appeared. This tendency to early strangulation makes femoral hernia dangerous. In 29 cases the tumor had existed from a few weeks to three years; in 1 its date was uncertain, and in 35 was more than four years; 27 years was the longest period recorded.

*Period of Strangulation.*—This was generally longer than in inguinal hernia, probably for two reasons: first, the reluctance of the women who are usually affected to apply for assistance; and, second, the comparatively small size of the tumor, causing it to be overlooked for a time.

| | | |
|---|---|---|
| 1st day of strangulation . . . . | 10 cases . . . | 1 death—from peritonitis. |
| 2d " " " | 20 " | 5 deaths—from cholera, internal strangulation, old bronchitis, previous peritonitis, and sloughing of the gut, each 1. |
| 3d " " " | 23 " | 6 deaths—from bronchitis, gangrene of gut, (2 cases,) ulceration and perforation (3 cases.) |
| 4th " " " | 11 " | 4 deaths—from exhaustion, peritonitis, (2 cases,) ulceration of gut. |
| 5th " " " | 9 " | 3 deaths—from peritonitis, gangrene of the gut, secondary hemorrhage. |
| 6th " " " | 4 " | 2 deaths—from peritonitis, ulceration of intestine. |
| Different days up to 10th . . . . | 6 " | 4 deaths—from exhaustion and peritonitis, (2 cases,) gangrene, ulceration of intestine. |

Two exploratory operations were also performed on patients dying with constipation and peritonitis; in one the sac was empty, and in the other the gut was not strangulated. It appears from the foregoing facts that the operation for femoral hernia, if early performed on a healthy patient, is free from any great risk.

*Truss.*—Strangulation is generally traceable to the neglect of wearing a well-fitted truss. Of the 85 cases, only 20 had worn a truss, and 2 of these only occasionally; 1 had only lately adopted it. The others wore either no truss at all, or a badly-made one.

*Contents of the Sac and their Condition.*—In 3 of the cases the sac was not opened; in 1 it was empty. Of the remaining 81, the sac contained

| | | | |
|---|---|---|---|
| Omentum alone in . . . | 5 | cases— | All recovered. |
| Healthy or congested small intestine in . . . . . . | 21 | " | 3 deaths. |
| Small intestine with effused blood in . . . . . . | 4 | " | 1 death from internal strangulation. |
| Inflamed small intestine in | 15 | " | 4 deaths. |
| Ulcerated, perforated, or gangrenous intestine, in . . | 8 | " | 5 deaths. |
| Small intestine with omentum— | | | |
| Both healthy, in . . . | 2 | " | Both recovered. |
| Intestine inflamed . . . | 5 | " | 3 deaths, 1 from cholera. |
| Omentum sloughy or consolidated . . . . | 9 | " | 3 deaths. |
| Intestine perforated or gangrenous . . . . | 3 | " | 3 deaths. |
| Omentum adherent . . | 4 | " | 2 deaths. |
| Omental sac " . . . | 4 | " | 2 deaths, 1 from bronchitis. |
| Large intestine . . . . . | 1 | " | Recovered. |
| | 81 | | 26 deaths, or 32·1 per cent. |

*Causes of Death.*—The 27 deaths from femoral hernia may be thus arranged:—

| | |
|---|---|
| Other diseases . . . . . . . | 3—viz.: cholera, internal strangulation, and bronchitis. |
| Exploratory operations . . . . . | 2 |
| Hemorrhage after operation . . | 1—probably from some small vessels. |
| Pure and simple peritonitis. . . | 6—in 2 cases it had existed before the operation; 2 of the other patients had had, one bronchitis and the other Bright's disease. |
| Exhaustion from protracted strangulation. . . . . . . . . | 2—it had lasted 7 and 8 days respectively. |
| Ulceration of the gut in the course of the stricture at the time of operation . . . . . . . . | 5 |
| The same, occurring subsequently. | 2 |
| Gangrene of the gut at the time of the operation . . . . . . . | 2 |
| The same, occurring subsequently. | 4 |

Hence it is inferred that the mortality of femoral hernia is due almost entirely to the negligence of the patients, and to the ignorance or apathy of their medical attendants; the rule at St. George's Hospital has for a long time been to operate very early.

*Sac opened or not.*—With respect to our tables alone we can draw no conclusions, by comparing the cases where the sac was opened with those where it was left intact; since with us it was opened in every case save four, and those of course were of the simplest character. But in the *Lancet* of 1856, there is a paper by Mr. Nathaniel Ward, in which he gives 100 cases from the London Hospital, where the rule is, *cæteris paribus*, not to open the sac; and as some deductions may be arrived at by comparing his table with ours, we have thought it advisable to make a similar classification of our cases and place them together, thus:—

*London Hospital.*

| No. of Cases. | Nature of Hernia. | | Sac opened. | Sac not opened. | Deaths. | |
|---|---|---|---|---|---|---|
| 100 | Umbilical | 4 | 4 | — | 3 | 33 per cent. |
| | Femoral | 63 | 21 | 42 | 19 | |
| | Inguinal | 33 | 24 | 10 | 11 | |

*St. George's Hospital.*

| No. of Cases. | Nature of Hernia. | | Sac opened. | Sac not opened. | Deaths. | |
|---|---|---|---|---|---|---|
| 121 | Umbilical | 3 | 3 | — | 1 | 30·5 per cent. |
| | Femoral | 85 | 82 | 3 | 27 | |
| | Inguinal | 33 | 32 | 1 | 9 | |

This comparison seems to show at least that very little additional risk is incurred by laying open the sac, while the information thus gained is certainly of no small importance.

The duration of the treatment in the favorable cases averaged for the inguinal variety thirty-six days, and for the femoral thirty-four.

On the question of opening a gangrenous intestine, or of leaving it alone, the slight evidence furnished by so small a number of cases is much in favor of making the opening; since, out of eight cases in which the intestine was found perforated or gangrenous, three recovered; and of these the gut was slit up at the operation in two instances. In one of these the artificial anus was nearly closed when the patient left the hospital, and eventually became entirely obliterated; in the other, the patient survived for more than a year with the artificial anus open, when, from some accidental cause, the adhesions between the gut and the parietes of the abdomen gave way, and the intestine was protruded and strangulated by the artificial anus. It was returned and reattached to the opening, but the patient sank.

II.—*On the Radical Cure of Inguinal Hernia.* By HOLMES COOTE, Esq., F.R.C.S., etc. (Lancet, June 1, 1861.)

Mr. Coote speaks of the case of a man in whom a strangulated hernia required operation, notwithstanding an attempt at the radical cure by Wützer's method, made three years previously, and with apparent success. Here, says Mr. Coote, "the inverted plug of integument had come down, and a fibrous constriction of the peritoneal sac remained, which proved the seat of the stricture. I believe this fibrous constriction, in part at least, to have been due to the changes consequent upon the passage of the needle in Wützer's operation, and it had served to keep up the intestine very fairly for above two years and a half. It then yielded, and became the seat of stricture to a protrusion somewhat larger than had ever been before."

Eight other cases are then adduced, in which inguinal hernia reappeared sooner or later after their radical cure had been attempted by surgeons of well-known skill and experience. Mr. Coote desires to call attention to the subject, with a view of testing the reliability of the procedure.

III.—*On Abdominal or Pelvic Abscess.* By F. C. SKEY, F.R.S., of St. Bartholomew's Hospital. (Dublin Medical Press, May 15, 1861.)

Mr. Skey ascribes this lesion, of which he has seen several instances during the last few months, to debilitating influences upon the constitution. Its general situation is the iliac fossa. "It appears in the form of a firm—not necessarily a hard—swelling, very distinctly perceptible on pressure over the above region. If small, its presence is only readily detectable by comparison with the opposite fossa, into which the ends of the fingers sink on moderate pressure. In this respect, however, there is a difference appertaining to the varieties in the form and quantity of the contents of the abdomen, especially of fat, and the greater or less laxity of the walls themselves. Occurring in young women shortly after parturition, its presence is remarkably distinct. When large, the swelling is apparent to the eye as well as to the touch—extending across the abdomen toward the mesial line, and upward in the direction of the umbilicus. In such cases the swelling is prominent, and as it increases in magnitude it encroaches on the intestines, which are pushed across to the opposite side. In many cases pain is not a prominent feature; and when present it is usually not severe, but is dull and aching rather than acute in character.

"In its early stage I have known this disease to be mistaken for two other varieties of swelling—malignant disease of the pelvis and scybalæ in the colon. From malignant disease it may be distinguished by the general uniformity of the swelling, and by the less serious constitutional signs of health undermined; and, judging from liabilities, malignant growths of or from the pelvis are far more uncommon than chronic abscesses. These examples of pelvic abscesses have presented themselves to my observation more commonly on the right than on the left side. I am not aware whether scybalæ collect more usually on that than on the left, but certainly they are more palpable and more readily detectable in the head of the colon than in the descending part of the intestine, which is placed in less proximity to the abdominal walls than that on the right side. But scybalæ are limited in their relation to the front abdominal walls by the caput coli, and, moreover, are movable; whereas the mass, which gradually resolves itself into the abscess, presents to the hand the sensation of a large and solid deposit, firmly fixed, and considerably larger than the intestine itself. The disease progresses very slowly, and often requires weeks for its development." Sometimes the abscess points directly through the skin, or it may follow the course of the femoral vessels, or pass backward through the sacro-ischiatic foramen; occasionally it extends across the abdomen behind the peritoneum, and so reaches the femoral vessels of the opposite side.

When the abscesses are large, of slow development, and so placed that the matter is bound down by the pelvic fascia, the subjacent bone is very apt to suffer. Several interesting cases in point are adduced.

The proper treatment of this lesion is "that which will most readily convert a chronic into an acute abscess. Any attempt to 'resolve' or 'discuss' these morbid deposits would be futile, and quite unworthy the advanced progress of scientific surgery. * * * * In order to obtain absorption of the deposited mass without passing into suppuration, we must convert the present stage of debility into the highest condition of vigorous health, and that is impossible. All that we can hope for, all that the best resources of art can achieve, is to change the chronic into an acute abscess, to advance the formation of pus, and to compel the abscess to select that locality through which it can most readily discharge its contents on a surface of the body. To effect this the appetite must be improved, and gratified with as large a quantity of nutritious food as can be digested; force and vigor must be given to the pulse by means of stimulants,—and the capacity for stimulants in these cases of debility is very great,—while the lungs should be supplied with an ample quantity of fresh air for the thorough oxygenation of the blood. If there be one therapeutic agent more valuable than another in promoting suppurative action it is bark, and it should be given throughout the treatment in full quantities. At the earliest moment at which fluid can be detected near the surface the abscess should be freely opened. It most commonly points through the abdominal muscles; but the rule equally applies should the abscess point toward the rectum, or when occurring in the female, toward the vagina, or on the nates or region of the trochanter."

IV.—*Cases of Gluteal Aneurism.* By MR. SYME. (Lancet, June 8th and 22d, 1861.)

In the first of these cases the aneurism had arisen spontaneously, in a young man otherwise healthy; it was of moderate size, pulsated freely, and seemed to affect the vessel at the point of its escape from the pelvis. Mr. Syme tied the internal iliac, in the usual way; the pulsation immediately ceased in the tumor, which lost two-thirds of its volume. The case did perfectly well, the ligature coming away on the seventeenth day, (not the twenty-fifth, as erroneously stated in one account.)

The other case was that of a middle-aged man, who was wounded by a pruning-knife driven deeply into his hip. The wound bled freely at the time, but became closed; subsequently the aneurismal tumor appeared, and at the time of the operation had attained the size of a man's head, its shape being bluntly conical.

"Mr. Syme commenced the operation by plunging a bistoury into the tumor. This was followed by a gush of arterial blood, which spouted to a considerable distance, but was immediately arrested by the operator introducing the forefinger of his left hand, the wound having been calculated for this. With his finger in the sac, Mr. Syme now explored the interior, hoping to discover the situation of the opening of the artery by the entering current. Nothing, however, could be felt but a confused mass of clots. The operator then enlarged the wound cautiously until the whole hand could be introduced, which was now thrust in up to the wrist. As the hand was being used to search the interior of the sac, it was so held that the wrist was nearly sufficient to occupy the wound; but during this procedure several alarming gushes of blood took place. * * * Compression of the abdominal aorta was tried; but as the patient lay nearly over on his face, this could only be done by pressing the abdomen up with one hand and the spine down with the other; and, as in John Bell's case, it appeared to do no good. Meanwhile, nothing satisfactory could be made out by the hand; only masses of irregular and very dense coagula could be felt, from among which the blood came welling up. At this time the situation appeared a very critical one, and to those who were unacquainted with the operator's resources it seemed that he had only to choose between standing there forever, plugging the wound with his hand, or to withdraw it and let the patient's blood gush out.

"Mr. Syme again took the knife, ran it swiftly up and down, laying freely open the whole cavity,* and scooped out the clots, one of which was of enormous size; the assistants, ready with sponges, darted them into the bottom of the sac, and the serious bleeding was at an end. All this was done with the greatest rapidity and precision, Mr. Syme stating afterward that, in taking this step, he had perfect confidence in his assistants, on whose intelligent aid so much depended. The bleeding from the artery could now be commanded by direct pressure; not, however, Mr. Syme stated, when made against the bone, but when made into the sciatic foramen. The mouth of the artery was now seized with artery forceps, and a ligature carried round it and tied. All bleeding and risk" [?] "were now at an end. The edges of the wound, which was directed downward and outward, were brought together and covered with a pledget of lint and a folded towel, but no bandage or compression was applied. The patient was under chloroform, and bore the operation well. The audience seemed surprised when Mr. Syme said that not much more blood had been lost than after a large amputation; but he reminded them that a large part of the blood that had escaped was that contained in the aneurism. In John Bell's case," [operated on at the same hospital sixty years ago,] "the patient had fainted from loss of blood, and was supposed to be dead before the sac was laid freely open. Mr. Syme also remarked that after the firm fibrinous coagula were removed, the whole of the cavity was found to be lined by a distinct and perfectly smooth membrane."

This case, like the preceding, has gone on favorably since the operation, as might indeed have been expected.

---

* The House Surgeon of the Infirmary, in a note published in the *Lancet* of June 29, says: "Before opening the sac further than to admit his hand, Mr. Syme separated all the dense layers of fibrin which he found firmly adherent to the bottom of the cavity, so that when a free incision was made by running the knife from one end of the tumor to the other, the whole contents, both solid and fluid, were evacuated instantaneously, and thus allowed the pressure of a finger to act at once directly on the bleeding orifice."

V.—*Aneurismal Varix in the Upper Part of the Thigh, following the employment of Pressure for the Cure of an Aneurism of the Posterior Tibial Artery.* Reported by OLIVER PEMBERTON, Esq., to the Royal Medical and Chirurgical Society. (Lancet, June 22, 1861.)

The patient, an old soldier, had suffered from aneurism of the posterior tibial artery for about three months. Pressure was made for three weeks upon the femoral artery where it passes over the pubis, and subsequently, for nine months, at a point below Poupart's ligament, just above the origin of the profunda; by which means a cure was effected. There was, however, some thickening where the pressure had been so long applied; and ten months afterward it became evident that a communication was established between the artery and the vein at that point. Eighteen months later, the man died from effusion into the pleural and peritoneal cavities, due to disease of the heart.

"The arterio-venous disease was found to be of that variety termed aneurismal varix. The right femoral artery and vein communicated by a large, distinct opening in the former vessel; while the vein was dilated and varicose, so as to have expanded itself into a sacculated covering for the greater portion of the artery in which the aperture had been established. There was no arterial aneurism, but the femoral artery was dilated at and above the aneurismal varix." Mr. Pemberton thinks that "the femoral vein became varicose from the pressure, causing obstruction to the return of its blood, and subsequently adhered intimately to the artery. Afterward, the communication was established between the two vessels, owing to the pressure of the varix on one hand, and to the action of the blood on diseased arterial coats on the other."

VI.—*New Mode of Reducing Luxations of the Shoulder-joint.* By Professor N. R. SMITH. (Am. Journal of the Med. Sciences, July, 1861.)

After reviewing the mechanical arrangements of the joint, the usual forms of its dislocation, and the difficulties in the way of their correction, Dr. Smith suggests that the most efficient counter-extension may be made from the wrist of the sound side, extension being effected from that corresponding to the injury. Having tested this idea for twenty years, he proposes a new plan of treatment, which he describes as follows:—

"I place the patient in a chair, sitting a little on one side of it, so as to allow room on the side of the injury for the operator's foot. I then pass a piece of stout muslin, folded, around the chest and under the axilla of the injured side. The tails of it I carry horizontally to the opposite side, one in front, the other behind, and extending the arm horizontally, bandage them firmly to the wrist of the sound side, leaving the ends projecting, to be well secured to the wall or other unyielding substance.

"I then pass an ordinary roller over the top of the injured shoulder, and back and forth, twice under the muslin band, to prevent its slipping down. Then I continue the same roller under the bottom of the chair and over the shoulder three or four times. This helps to give steadiness to the scapula, and especially to prevent the involuntary rising of the patient from the chair, or the tilting of the scapula upward, when it is necessary to make the manipulation of which I am to speak.

"I now attach the extending band to the wrist of the injured side. * * * * I first apply a wet roller to the wrist, and then attach a muslin band by the clove hitch. Next I direct the extension to be made by two persons, at first outward and a little downward, gradually raising the arm to the horizontal direction, and finally a little above it. The extension must be made gently and steadily, gradually increasing the force, so as not to provoke the muscles to spasmodic resistance. As no pain is created by the force thus employed, it may be continued for a considerable time. The muscles, which at first resist, become fatigued, and finally relaxed, and, in a large majority of instances of recent luxation, the head will slip into place without resort to any species of manipulation. I would even continue this traction, where much resistance is encountered, for a quarter of an hour before modifying the force; but in case the object is not then effected, let the surgeon place his foot on the margin of the chair, and his knee in the axilla. Then let the assistants raise their line of traction above the horizontal as much as possible, and continue it for a moment. The surgeon should then direct that the arm be, by a sudden movement, carried downward, while, by extending his foot, he elevates the knee in the axilla. He aids the assistants in this by grasping the arm near the elbow and using it as a lever. * * * * Where the consecutive displacement under the coracoid has occurred, the procedure is nearly the same, except that I make the traction a little more in the direction backward and upward, so as to disengage the head from under the process."

With regard to the dislocation upon the dorsum scapulæ, Prof. Smith says that he has seen but three cases, and in only one of these was an attempt at reduction warranted. "I failed," says he, "to effect the reduction in the method usually recommended, and which I had deemed the best. I did not then practice extension from the opposite wrist. I made it by carrying the arm through a slit in a sheet. The extension was made from the wrist, and when continued for some time, I sought to throw the head into place by manipulation—that is, I placed my knee against the back of the neck of the bone, and swinging the arm backward, endeavored to prize the head of the bone into its place. I repeated the effort several times, but with no satisfactory result.

"I then carried a band over the front of the shoulder, one tail under the axilla, the other above it. These I united, carried them backward and inward obliquely, and secured them to the wall. Then I made traction strongly from the wrist almost directly forward. Without much difficulty I thus drew the head of the bone forward over the margin of the glenoid, and had the satisfaction to see it slip into its place."

VII.—*Anterior Suspensory Apparatus for the Treatment of Fractures of the Lower Extremity.* By Prof. N. R. SMITH. (Am. Journal of the Med. Sciences, April, 1861.)

Dr. Smith defines the objects to be aimed at in treating fractures of the lower extremity as follows: "1st. To furnish a surface of support which shall be accurately and permanently adapted to the surface and form of the limb which reposes in it. 2d. To so adapt the surface of support as that the limb, sinking into it, shall maintain its form by its own weight, without the necessity of lashings here and there, to secure it to the rigid portions of the apparatus. 3d. To make the limb obedient to all the unavoidable movements of the trunk, so that the upper fragment shall not be jammed upon the lower, nor contorted in relation to it by the movements of the trunk. 4th. To obviate the contraction of the muscles by the employment of an extending force, uniform in its action, easily graduated, and not requiring the application of bands to the ankle. 5th. So to arrange the supports that in compound fractures we may have free access to the seat of injury, without removing any of the essential supports of the limb. 6th. To effect these objects by an apparatus simple in construction, capable of being anywhere procured, easy of application, not requiring to be readjusted, and of but trifling expense."

We have not room here to quote the whole of Dr. Smith's interesting article, but must be content with giving his description of his apparatus. The splint, as now used by him, is made of wire, about as thick as a No. 10 bougie. If lighter than this, it is apt to spring too much. One piece of wire is bent twice at right angles at each extremity, so as to form a very long parallelogram, three inches wide at its upper extremity, two-and-three-quarters at its lower. "It must be long enough to extend from a point a little above the anterior spinous process of the ilium, to an inch beyond the toes when the leg, foot, and thigh are extended. Three feet eight inches will be sufficiently long for most adults. A little excess of length, above or below, is unimportant. The side-pieces are to be sustained by cross-pieces at distances of about eight inches, firmly clinched upon them. The wire frame is then to be bent by the surgeon to suit the case. The lower angle corresponds to the ankle, and is one of about 120°, to secure an easy posture of the foot. The angle at the knee is very obtuse—about 160°. The angle at the hip should be about the same degree. The angle at the ankle should be about six inches from the extremity; that of the hip seven. The middle bend corresponds to the knee. The angles are easily made by bending the splint over the margin of a strong chair or table. It will often be necessary to vary these angles to suit particular fractures. The wire frame is now to be tightly wrapped in a muslin bandage, and it is ready for application.

"The suspensory apparatus is simple and easy of application. A small iron pulley is to be screwed into the ceiling, over the bed of the patient, perpendicularly over the middle of the shin, or nearly so. A cord, about as thick as the wire of the splint, passes over the pulley and is reeved through a small tent-block, by which, slipping it upward or downward, we elevate or depress the limb. The eccentric pressure prevents the weight of the limb from causing it to slip; if not, rub the cord with chalk. This single cord, which depends from the block, has a loop at its end, about two feet or more above the limb. Through this another cord, about five feet long, passes and hangs double from the loop, by its centre. Each end has a double hook attached to it. It is made of much smaller wire, has an expansion nearly equal to the breadth of the splint, and has the points sharpened and abruptly turned inward, the bends being quite angular, so as snugly to embrace the wire and not slip upon it. When everything is ready for the suspension, these hooks are to be applied to the wire-frame, piercing with them the muslin cover of the splint."

The splint being applied along the front of the limb, the latter being supported by assistants, strips of starched bandage are arranged at different points so as to suspend the limb from the splint. The hooks are then applied, and the cord pulled upon so as to swing up the splint, and with it the limb. If the splint does not fit, it may be made to do so by changing the position of the hooks. A roller is now applied to the whole limb, finishing with a spica of the groin and one or two direct turns around the pelvis. Sometimes the whole under portion of this bandage may be smeared with starch. Extension and counter-extension are now made at once, by moving the bed so as to render the cord slightly oblique; if necessary this may be rendered more effectual by putting blocks under the footposts, so as to raise the lower end of the bed. The heel should just hang clear of the surface below it; a small pillow may be so placed as to steady the upper part of the thigh.

Such is the mode of application of this method to fractures of the thigh; it needs some modifications which will readily suggest themselves, when employed in fractures of the other bones of the lower extremity.

